# The First-in-Human Synthetic Glycan-Based Conjugate Vaccine Candidate against *Shigella*

**DOI:** 10.1021/acscentsci.1c01479

**Published:** 2022-03-17

**Authors:** Robert M. F. van der Put, Carolien Smitsman, Alex de Haan, Martin Hamzink, Hans Timmermans, Joost Uittenbogaard, Janny Westdijk, Michiel Stork, Olga Ophorst, Françoise Thouron, Catherine Guerreiro, Philippe J. Sansonetti, Armelle Phalipon, Laurence A. Mulard

**Affiliations:** † Intravacc, P.O. Box 450, 3720 AL Bilthoven, The Netherlands; ‡ Institut Pasteur, U1202 Inserm, Unité de Pathogénie Microbienne Moléculaire, 28 rue du Dr Roux, 75724 Paris Cedex 15, France; § Institut Pasteur, Université Paris Cité, CNRS UMR3523, Unité de Chimie des Biomolécules, 28 rue du Dr Roux, 75724 Paris Cedex 15, France; ∥ Chaire de Microbiologie et Maladies Infectieuses, Collège de France, 11, place Marcelin Berthelot, 75005 Paris, France

## Abstract

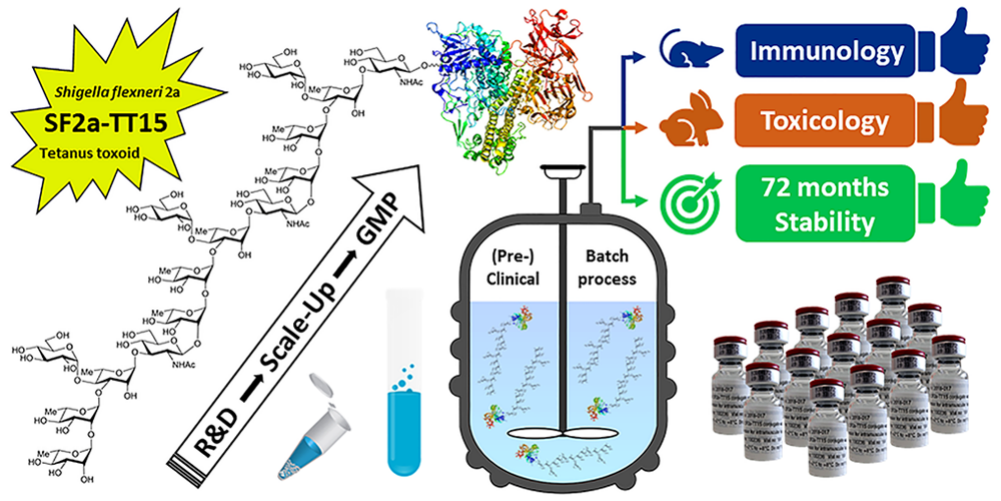

*Shigella*, the causative agent of shigellosis, is among the main causes of diarrheal diseases with still a high morbidity in low-income countries. Relying on chemical synthesis, we implemented a multidisciplinary strategy to design SF2a-TT15, an original glycoconjugate vaccine candidate targeting *Shigella flexneri* 2a (SF2a). Whereas the SF2a O-antigen features nonstoichiometric O-acetylation, SF2a-TT15 is made of a synthetic 15mer oligosaccharide, corresponding to three non-O-acetylated repeats, linked at its reducing end to tetanus toxoid by means of a thiol-maleimide spacer. We report on the scale-up feasibility under GMP conditions of a high yielding bioconjugation process established to ensure a reproducible and controllable glycan/protein ratio. Preclinical and clinical batches complying with specifications from ICH guidelines, WHO recommendations for polysaccharide conjugate vaccines, and (non)compendial tests were produced. The obtained SF2a-TT15 vaccine candidate passed all toxicity-related criteria, was immunogenic in rabbits, and elicited bactericidal antibodies in mice. Remarkably, the induced IgG antibodies recognized a large panel of SF2a circulating strains. These preclinical data have paved the way forward to the first-in-human study for SF2a-TT15, demonstrating safety and immunogenicity. This contribution discloses the yet unreported feasibility of the GMP synthesis of conjugate vaccines featuring a unique homogeneous synthetic glycan hapten fine-tuned to protect against an infectious disease.

## Abstract 2

A conjugate vaccine featuring a unique synthetic glycan hapten induces protective antibodies to most **Shigella flexneri** 2a circulating strains: GMP process, successful toxicology data, and long-term stability.

## Introduction

**Figure 1:**
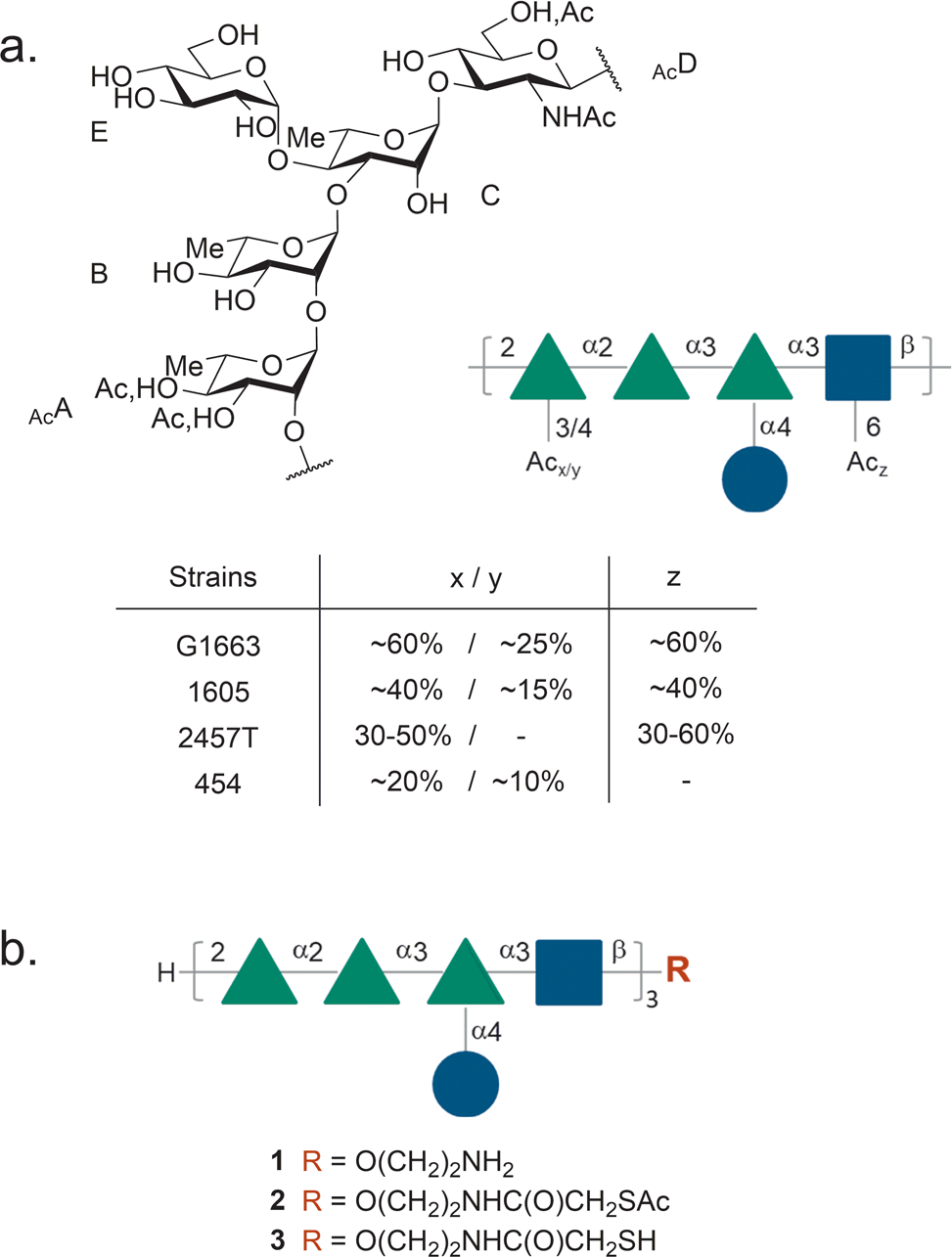
(a) Repeating unit from the SF2a O-Ag showing the sites and ratios of nonstoichiometric O-acetylation.^2−4^ (b) Structure of the 15mer oligosaccharide identified as an antigenic, structural, and conformational mimic of the SF2a O-Ag in the form of its aminoethyl glycoside (**1**, [AB(E)CD]_3_-NH_2_) and equipped with a conjugation-ready linker featuring a masked thiol moiety (**2**, [AB(E)CD]_3_-SAc) or a thiol moiety (**3**, [AB(E)CD]_3_-SH). Solid green triangles: l-rhamnopyranose; solid blue squares: *N*-acetyl-d-glucosamine; solid blue circles: d-glucopyranose.

Bacillary dysentery, or shigellosis, is associated with a significant burden globally. With more than 250 million annual cases estimated to occur in low- and middle-income countries,^5^ it is one among the four most prevalent diarrheal diseases, affecting in particular children less than five years of age.^6,7^ In this population, frequent diarrheal episodes have been correlated to long-term growth and cognitive impairments.^8^ In adults, shigellosis has a higher incidence in the elderly^5^ and is a well-established cause of diarrhea in travelers and military personnel.^9^ Antimicrobial resistance is growing, which reduces opportunities for efficient treatment,^10,11^ and contributes to enhance concern whether from the CDC^12^ or the WHO.^13^ Improved sanitation and access to clean water represent effective means of preventing shigellosis, but they qualify as a lengthy process. In this context, the development of a *Shigella* vaccine suitable for use in children under the age of five living in low- and middle-income countries is highly desirable.^14^ In particular, such a vaccine should provide protection against *Shigella flexneri* and *Shigella sonnei*.^15^*Shigella* lipopolysaccharide (LPS) is an important virulence factor,^16^ and its O-antigen (O-Ag) component is a major protective antigen.^17,18^ On the basis of the assumption that serum antibodies to the *Shigella* O-Ag could protect against reinfection,^19^ and pioneered by Drs. J. B. Robbins and R. Schneerson (NIH, MD, USA),^20^ several parenterally administered detoxified LPS-protein conjugate vaccine candidates have been developed and tested in clinical trials.^21^ Protective efficacy was demonstrated for a *S. sonnei* detoxified LPS-rEPA (recombinant exoprotein from *Pseudomonas aeruginosa*) vaccine prototype in adults and children older than three years of age, although not in the youngest vaccine recipients.^22,23^ Leading the path forward, these prime inspiring achievements prompted further studies on alternatives to this first generation of *Shigella* polysaccharide–protein conjugate vaccines.^21^ An increasing interest in vaccines to fight infectious diseases, enhanced in a context of the fast emergence of antibiotic resistance,^24−27^ and an improved, albeit still limited, understanding of their mechanism of action^28−31^ contribute, among other factors, to trigger major developments in the field of glycoconjugate vaccines.^32−34^ Otherwise, compelling evidence substantiates the identification of serum IgG antibodies to *Shigella* LPS as a good correlate of protection against shigellosis.^35^ In this context, novel families of LPS-based *Shigella* vaccines have successfully passed phase 1 clinical trial and more.^36−38^ Synthetic glycans for use as LPS surrogates have been the subject of special interest. They were actively investigated in the search for improved vaccine candidates able to induce anti-LPS serum IgG titers suitable for protecting children under three years of age against shigellosis.^21^ In the late 1990s, groundbreaking studies on *S. dysenteriae* 1 showed the superiority in mice of synthetic oligosaccharide-based sun-type conjugates over lattice-type conjugates issued from the random conjugation of the detoxified LPS to rEPA.^39^ Aiming at defeating shigellosis, in particular, *S. flexneri* and *S. sonnei*, which account for some 66% and 24% of the global *Shigella* burden, respectively,^15^ a related multidisciplinary glycochemistry-based strategy was implemented at Institut Pasteur.^40−45^ The most advanced work concerns *S. flexneri* 2a (SF2a), the prevalent *S. flexneri* serotype.^15^ The SF2a O-Ag is defined by a branched pentasaccharide repeating unit O-acetylated in a nonstoichiometric manner at two sites (Figure 1a).^2−4^ A chemical biology strategy^46^ involving extensive epitope mapping was implemented, which led to the identification of a synthetic pentadecasaccharide [AB(E)CD]_3_-NH_2_ (**1**)^1^ corresponding to three non-O-acetylated O-Ag repeating units as an antigenic,^47^ conformational,^4^ structural,^48^ and functional mimic^49^ of the SF2a O-Ag (Figure 1b). Moreover, the well-defined synthetic O-Ag segment **1** was recognized by sera from naturally infected individuals.^49^ A glycoconjugate issued from the single-site attachment of [AB(E)CD]_3_ to tetanus toxoid (TT), a medically acceptable carrier, by means of a chemoselective conjugation between the thiol-equipped **3** and maleimide-equipped tetanus toxoid (TT_Mal_), was shown to induce high anti-SF2a O-Ag IgG antibody titers in mice.^49^ In addition, an adjuvanted B,T-diepitope glycoliposome displaying the synthetic [AB(E)CD]_3_ hapten was shown to induce a proper anti-SF2a immune response.^50^ These findings support our original assumption that the well-defined synthetic **1** featured a promising surrogate of the highly heterogeneous natural SF2a O-Ag.

**Scheme 1:**
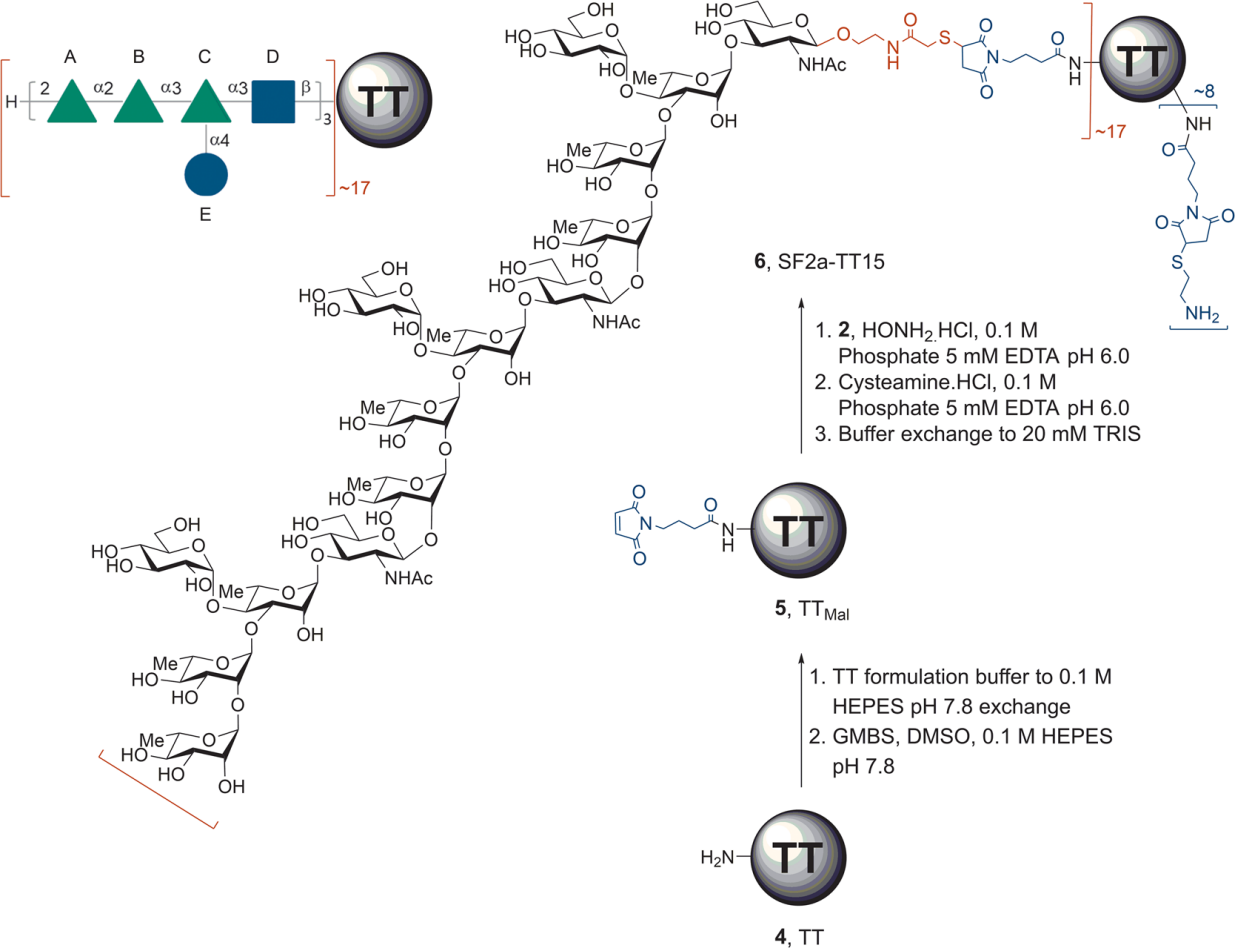
SF2a-TT15 (**6**, [AB(E)CD]_3_-TT), a Vaccine Candidate against SF2a and Synthetic Process to Achieve Its Production from TT (**4**) and the Linker-Equipped Oligosaccharide **2** GMBS: *N*-[γ-maleimidobutyryloxy]succinimide ester, HEPES: 4-(2-hydroxyethyl) piperazine-1-ethanesulfonic acid, TT: tetanus toxoid, TT_Mal_: maleimide-modified tetanus toxoid.

Featuring strong immunogenicity in mice and synthetic manufacturing feasibility, an [AB(E)CD]_3_-TT conjugate characterized by an average oligosaccharide:TT molar loading of 17 ± 5—SF2a-TT15 (**6**, Scheme 1)—was subsequently identified as a promising SF2a vaccine candidate to move forward for evaluation in humans.^51^ SF2a-TT15 is obtained according to a three-step conjugation process from the chemically synthesized linker-equipped oligosaccharide **2** bearing a masked thiol and commercially available TT (**4**) (Scheme 1).^49^ Thus, TT_Mal_ (**5**), resulting from the grafting of 4-maleimidobutyric acid *N*-hydroxysuccinimide ester (GMBS) on TT (step 1), reacts with conjugation-ready **3**, issued from the selective unmasking of the thiol moiety in the more stable precursor **2**, in a precisely controlled **2**:**5** ratio (mol:mol) of 25 (step 2). Cysteamine-capping of the unreacted maleimide moieties in the conjugation product (step 3) then provides glycoconjugate **6**. Relying on an exhaustive design of experiments (DoE) study aimed at assessing key parameters (temperature, pH, concentration, reaction time, GMBS/TT ratio) to warrant optimal oligosaccharide loading and minimal aggregation, a robust, high yielding chemoselective thiol-maleimide conjugation procedure was established at microscale.^51^

Herein, the scale-up and implementation of this microscale conjugation process in a format complying with good manufacturing practice (GMP) conditions, by use of GMP-grade [AB(E)CD]_3_-SAc precursor **2** (^GMP^**2**) and TT (^GMP^**4**), are reported. A preclinical batch and a clinical batch of SF2a-TT15, each accounting for tens of thousands of doses of the vaccine candidate, were produced to perform the first-in-human clinical trial, including the requested repeated-dose toxicity study. As part of the preclinical requirement for the latter, we also report on the immunogenicity in both mice and rabbits, and provide extended stability data in support of the shelf life for the formulated SF2a-TT15 vaccine candidate. Moreover, we demonstrate for the first time that SF2a-specific antibodies elicited upon immunization in mice with a fine-tuned SF2a glycoconjugate vaccine prototype, made of a well-defined chemically synthesized non-O-acetylated O-Ag segment, display *in vitro* bactericidal properties and are able to recognize a large diversity of SF2a strains circulating in different geographical settings. Lastly, the requirements for a broad serotype-coverage *Shigella* vaccine are discussed.

## Results and Discussion

Licensed conjugate vaccines are routinely administered in minute amounts, roughly corresponding to 1–10 μg type- or group-specific glycan per vaccine dose.^52^ Available preclinical data supported the assumption that SF2a-TT15 obeys the same rule. This observation had a direct consequence on the GMP process to be developed. It guided the production scale, and therefore the equipment selection, among which were the reactor and filtration device. To fulfill GMP-grade criteria, in particular, relevant to impurity content and scalability, all filtration steps were performed by tangential flow filtration (TFF) instead of spin filtration as originally described.^51^ Owing to limitations in terms of the minimal volume compatible with this technology, a 1 L reactor was—at that time—the smallest commercially available vessel found suitable to fulfill the above-mentioned criteria. It was subsequently used specifically during the process development steps regarding scale-up studies and evaluation of process performance with respect to impurity removal in the absence of the thioacetate precursor **2**. It is worth mentioning that, irrelevant to the clinical demand, in this case the GMP production scale of both the preclinical batch and clinical batch of SF2a-TT15 had to be adapted to comply with the limitations of the commercially available GMP equipment.

### Process Development

**Figure 2:**
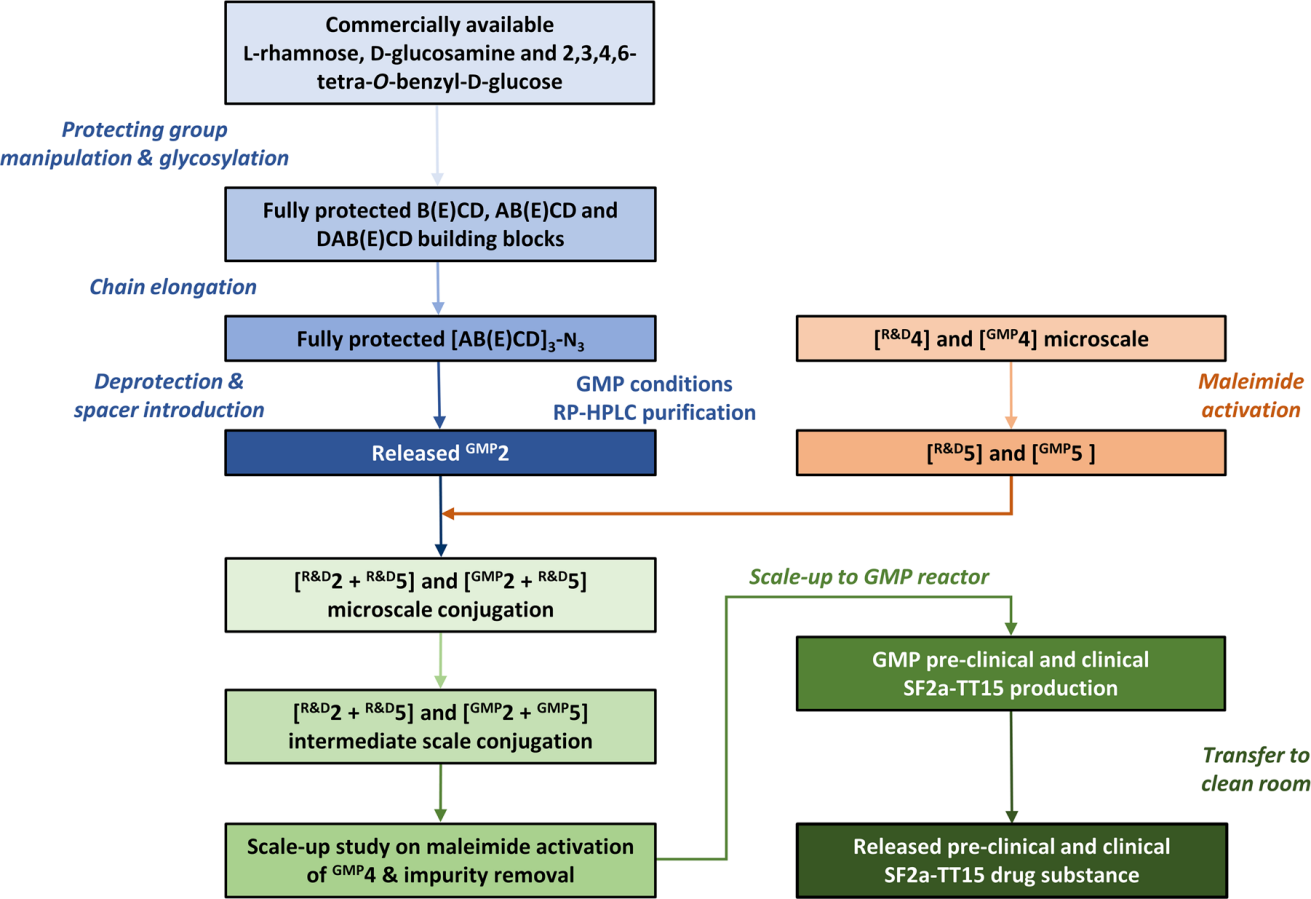
Overview of process development and SF2a-TT15 bulk release. Blue panels: synthesis of GMP-grade precursor **2** (^GMP^**2**).^1^ Orange panels: maleimide activation of **4** and conjugation reaction with the introduction of **2**. Green panels: process development for GMP manufacturing of SF2a-TT15 from precursor **2**. Release of the (pre)-clinical conjugate vaccine DS was based on impurity assessment (NMR), free and total carbohydrate content (HPAEC-PAD), osmolality, pH, endotoxin, and aggregate content (HPSEC). HPAEC-PAD: high-performance anion-exchange chromatography with pulsed amperometric detection, HPSEC: high-performance size exclusion chromatography.

With our previous achievements serving as a ground basis,^1,51^ we engaged in the process development to GMP manufacturing (Figure 2). In the first place, GMP-grade [AB(E)CD]_3_-SAc (^GMP^**2**) was produced on the multigram scale (Figure 2, not described). In brief, the multistep chemical process was adapted from an established route^1^ to reach the known fully protected [AB(E)CD]_3_-N_3_ intermediate, which was then converted in four steps to thioacetate **2** under conditions fulfilling GMP criteria. The GMP-grade material, or drug substance (DS) intermediate, was released based on the evaluation of the certificate of analysis and ^1^H NMR analysis for identity and purity (Figure S1). The total amount of carbohydrate was assessed by high-performance anion-exchange chromatography with pulsed amperometric detection (HPAEC-PAD). Further process development for the final scaled-up (pre)-clinical DS is described below.

### From Microscale to Intermediate-Scale Bioconjugation

Having the key oligosaccharide precursor ^GMP^**2** in hand, the reaction kinetics and the efficiency of the conjugation step at microscale (0.16 mL, 0.02 μmol of TT) were confirmed using research-grade TT (^R&D^TT) on the one hand and both research-grade and GMP-grade oligosaccharide **2** (^R&D^**2** and ^GMP^**2**) on the other hand. As expected, the final [AB(E)CD]_3_:TT loading (mol:mol) was fully controlled in a reproducible manner, simply based on the amount of the masked thiol **2** engaged in the reaction (Table S1, Figure S2). The source of the hapten precursor had no detectable influence.

At the intermediate scale (1.0 mL, 0.13 μmol of TT), the modification step performed equally well for ^R&D^TT as for ^GMP^TT with respect to aggregate induction (<7.5%) and activation efficiency (>80%). The conjugation step *per se* was investigated for ^GMP^TT under the conditions established to achieve an [AB(E)CD]_3_:TT loading of 17 ± 5 (mol:mol), as required in the targeted SF2a-TT15. Satisfactorily, the use of 25 mol equiv of ^GMP^**2** per ^GMP^**5** resulted in an average [AB(E)CD]_3_:TT loading of 17 ± 1 (mol/mol) corresponding to a conjugation efficiency of 68 ± 4% and a yield of 62 ± 5% over two steps from ^GMP^**2** (Table S1). As expected, the HPSEC profiles were very similar to those of the corresponding microscale experiments. The demonstration that process scale-up had no influence on reaction kinetics (Figure S3) or on the conjugate HPSEC profile supported a deep investigation of impurity removal.

### Impurity Removal and Process Performance

As part of this fourth step, a profile was established for each of the impurities present in the bulk vaccine candidate. The fact that the concentrations of impurities were undetectable in the final drug product (DP) led to the rationale for measuring impurities in the DS bulk instead. The maximum allowed concentration of each one of those impurities in the bulk vaccine was calculated from the respective specifications for final products in the ICH guidelines (Table 1). Interestingly, the starting concentrations of DMSO, cysteamine·HCl, and acetohydroxamic acid were already significantly lower than the maximum concentrations allowed according to respective guidelines (Table 1). However, the removal of these impurities was still evaluated to confirm process performance. The latter was primarily investigated by mimicking the large-scale process with only buffers and excipients. Impurity removal was evaluated by comparing the theoretical starting concentrations of excipients and impurities with the actual starting concentrations and decline thereof during each filtration step and compared to specifications set (Table 1). Single-use filters were favored so that a full cleaning validation necessary for GMP production could be omitted. Two filters, selected for their resistance to DMSO (40%) and to hydroxylamine (2 M), were assayed. They differed.

**Table 1:** Impurity Considerations during Scale-up of the TT Modification and Conjugation Steps in the Absence of Oligosaccharide **2** and TT

impurity	guideline for GMP	maximum allowed quantity in the formulated vaccine (μg/dose or μg/0.5 mL unless indicated otherwise)	maximum allowed quantity in the bulk vaccine^a^ (mM)	estimated maximum concentration in the bulk vaccine (mM)
GMBS/GMBA	ICH-M7	12	<9	15.2^b^
*N*-hydroxysuccinimide	ICH-M7	12	<9	15.2^b^
DMSO	ICH-Q3D	5000 ppm	<6400^c^	761^d^
hydroxylamine·HCl	ICH-M7^draft status^	2	<12	37.4^e^
acetohydroxamic acid^f^	n.a.	72	<73	2.5^g^
EDTA^h^	ICH-M7	12	<6	5^h^
cysteamine·HCl^i^	n.a.	200	<518	14.4^j^

a The bulk SF2a-TT15 is ≥100 times more concentrated than the formulated vaccine candidate.

b *N*-Hydroxysuccinimide is a byproduct resulting from GMBS coupling to TT or from its hydrolysis into GMBA. Here, we assume 100% formation of byproduct.

c 2019 specification for DMSO is 6400 mM, which corresponds to 5000 ppm per vaccine dose.

d [(volume DMSO mL/total volume mL) × 1000 = g/L]/*M*_w_ (DMSO) g/mol thus (6.33/106.33) × 1000/78.13.

e (0.26 g/100 mL) × 1000 mL/*M*_w_: 69.49 g/mol.

f Acetohydroxamic acid is used as a treatment for bladder infections. A normal dose is 12 mg/kg/day, which translates to 720 mg/day for a 60 kg adult. When applying a minimum 1000-fold decline with respect to process performance and an additional 10-fold safety margin, a maximum amount of 1/10000 of the starting concentration of thiol **3** is acceptable.

g 6.47 mg/mL: same concentration as thiol **3**.

h The concentration of EDTA in conjugation buffer is 5 mM.

i Cysteamine·HCl is administered as a treatment for nephropathic cystinosis. A daily dose of 2 g/day is accepted. When applying a minimum 1000-fold decline with respect to process performance and an additional 10-fold safety margin, a maximum amount of 1/10000 of initial concentration is acceptable or 200 μg/dose.

j (180 mg/110 mL total) *1000 mL/Mw: 113.61 g/mol. ICH: International Council for Harmonisation of Technical Requirements for Pharmaceuticals for Human Use by their flow paths, LP screen and open channel, respectively. GMBA: 4-maleimidobutyric acid.

Simulating the maleimide activation of **4**, conjugation of the resulting **5**, and capping of the conjugation product **6** has given insight in process performance of the two different filters (see Supporting Information). Both filters performed equally in terms of impurity removal, except for DMSO. Extraction of DMSO during simulation of the conjugation reaction yielded 50% lower concentrations for the LP screen filter (Figure S4). In addition, during final purification, DMSO was removed more efficiently using the LP screen filter, and the full array of impurities was removed to below 0.1% of their respective starting concentrations (see Figure S3 and Table 2). Furthermore, higher filtrate flows and lower transmembrane pressure (TMP), inlet and outlet pressures observed during processing (not discussed) led us to select the LP screen filter for GMP production.

**Table 2:** Impurity Removal When Using the LP Screen Filter

impurity^a^	maximum Q (mM)^b^	BEV^c^	removal below spec. (yes/no)
GMBS	<9	6	yes
GMBA	<9	6	yes
*N*-hydroxysuccinimide	<9	6	yes
DMSO	<6400	4	yes
hydroxylamine·HCl	<12	8	yes
cysteamine·HCl	<518	6	yes

a No data available for acetohydroxamic acid and EDTA.

b Maximum allowed quantity (*Q*) in the bulk vaccine (mM). See Table 1.

c Amount of buffer exchange volumes needed for 99.9% removal.

### Scale-up Study on Maleimide Activation of TT

At production scale (100 mL), the buffer exchange and subsequent concentration of ^GMP^**4** to 1.07 mM, followed by its reaction with GMBS, yielded very similar results as compared to previous microscale experiments. The amine content (mol/mol) of ^GMP^**5** and the amount of modified amines (mol/mol) derived thereof were 3.3 and 20.9, respectively. Likewise, the amount of aggregates fulfills the established threshold of <7.5% (Table 3).

**Table 3:** Scale-up of the Maleimide Modification of **4** into **5**

	microscale^a^^,^^b^	production scale^b^
amine/protein molar ratio in **4** post-buffer exchange	27.1 ± 1.9	29.5
amine/protein molar ratio in **5**	5.1 ± 2.0	3.3
total amount of modified amines in **5**	22.1 ± 0.6	20.9
amount of aggregates in concentrated **4** (%)	29.9 ± 3.4	30.1
amount of aggregates in **5** (%)	36.8 ± 3.8	33.9
aggregate induction (%)^c^	7.0 ± 3.0	3.8

a Microscale experiments results show average and 1 × standard deviation (SD) (*n* = 5).

b ^GMP^TT used for both preclinical and clinical batches was of the same lot.

c Aggregate induction = amount of aggregates in **5** (%) – amount of aggregates in concentrated **4** (%).

### GMP Production of Preclinical and Clinical Batches of SF2a-TT15

The GMP production process was defined using the results of the large-scale ^GMP^**4** to ^GMP^**5** conversion, impurity removal investigation, and micro- and intermediate-scale bioconjugation. Reactant ratios for the maleimide introduction, conjugation, and capping steps were kept similar as in the micro- and intermediate-scale experiments. However, two changes in the purification strategy were applied. The amount of buffer exchange volume (BEV) was reduced from 10 to 5 for the purification of ^GMP^**5** and of ^GMP^**6** in order to reduce the process time and buffer usage. Nevertheless, final buffer exchange after capping remained at 10 BEV to ensure that all impurities were removed below the criteria set. This yielded preclinical and clinical batches comprising on average 19 and 17 oligosaccharide chains per TT, respectively, which was well within specifications (17 ± 5). Furthermore, the concentration of all impurities was well below the specifications set (Table 2). The yield of preclinical and clinical bulks was 76% and 80%, respectively, based on the starting amount of oligosaccharide ^GMP^**2**, weighted and corrected for water content, and final carbohydrate content in the bulk. This remarkable achievement supports the robustness and scalability of the newly established GMP single-site conjugation process.

Final fill and finish was achieved in 20 mM TRIS·HCl containing 150 mM NaCl, to yield two formulations of the SF2a-TT15 conjugate vaccine candidate corresponding to a 2 μg and 10 μg amount of [AB(E)CD]_3_ oligosaccharide per dose (0.5 mL), respectively, based on an HPAEC-PAD quantification assay of saccharide content. Both formulations complied to all set specifications and were subsequently included in a stability study.

In addition, the 10 μg formulation was used in a toxicology study and exhaustive immunogenicity study.

### Stability of the SF2a-TT15 Preclinical and Clinical Batches

**Figure 3:**
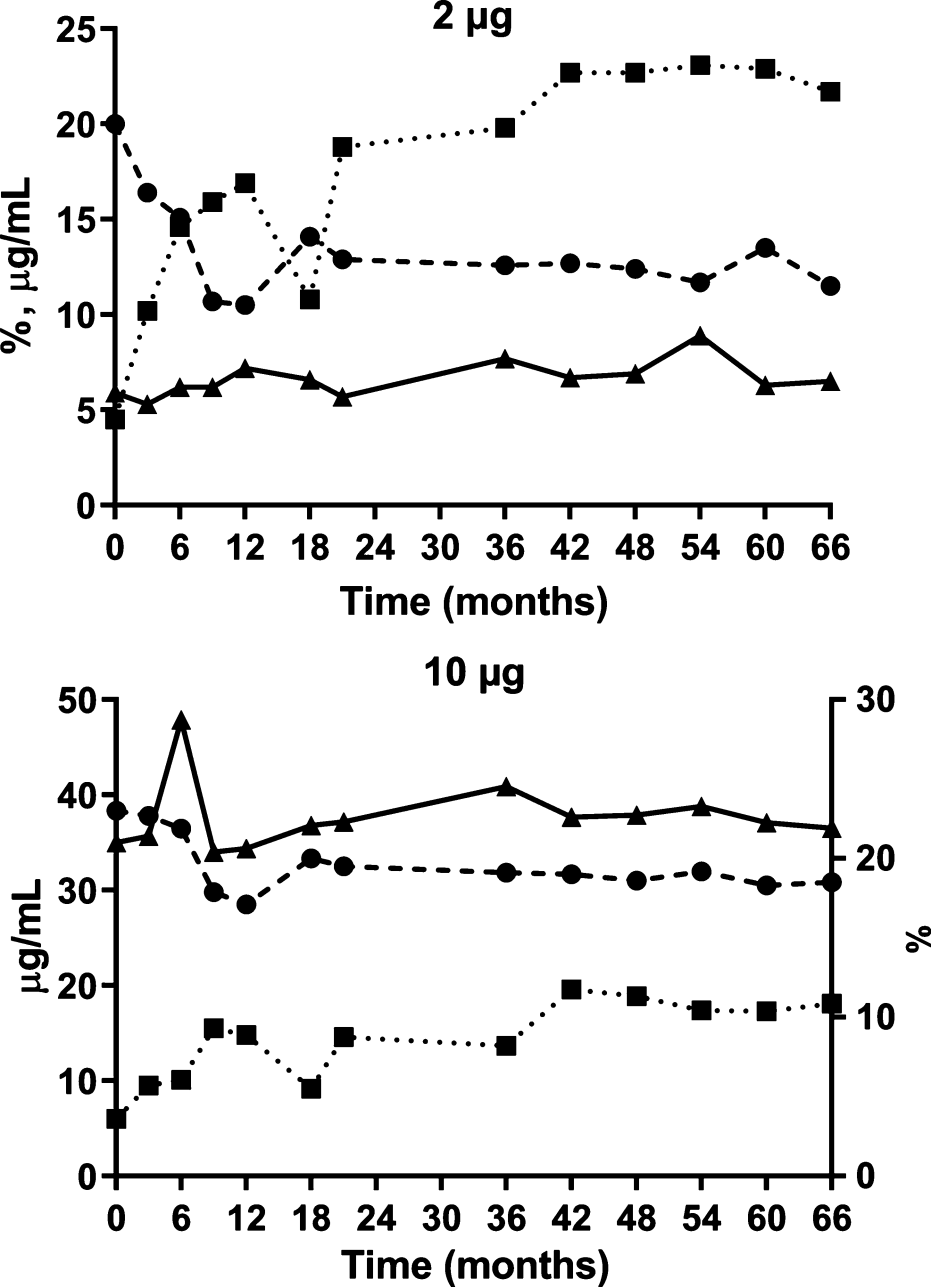
Real-time stability of the SF2a-TT15 clinical batch for two different formulations. Top panel: 2 μg carbohydrate equivalent per vaccine dose (amount of SF2a-TT15 corresponding to 8 μg of carbohydrate per mL). Bottom panel: 10 μg of carbohydrate equivalent per vaccine dose (amount of SF2a-TT15 corresponding to 40 μg of carbohydrate per mL). *x*-axis: Time (months), (●) aggregation (%, average of two replicates); (■) free carbohydrate content (% of total carbohydrate, average of three replicates, SD < 0.1%); (▲) total carbohydrate content (μg/mL, average of three replicates, SD < 0.1%). SD: standard deviation (not shown).

A real time stability study was initiated, where both formulations (8 and 40 μg/mL oligosaccharide, after a two-fold dilution yielded the 2 and 10 μg oligosaccharide equivalent per vaccine dose (0.5 mL)) were stored at 2–8 °C and evaluated at different time points (Figure 3). Stability was assessed by evaluating visual aspects, osmolality, pH and protein content (data not shown), aggregation (molecular size distribution), as well as free and total carbohydrate content.

With respect to aggregation, we observed a downward trend in the first nine months for both formulations in which a root cause analysis was attributed to the analysis and not to a change in the composition of the formulated SF2a-TT15. No significant changes were observed during the remainder of the stability study.

The free carbohydrate content started at 4.1% for the bulk before final fill and finish, for both preclinical and clinical batches. When this parameter was assessed after fill and finish, a significant increase was detected during the first 12 months of conservation, from 4.5% and 6.0% to 16.9% and 14.8% for the vials corresponding to 2 μg and 10 μg oligosaccharide equivalent per vaccine dose, respectively. While existing, this increase was subsequently much less perceivable to reach 19.8% and 13.7% at 36 months, and 21.7% and 18.1% at 66 months, respectively. All parameters taken into account, the real-time stability data of the preclinical batch demonstrated product stability for a period of at least 66 months, which was exceptional since according to current ICH guidelines (ICH-Q1-A-(R2)) a 12-month period would have sufficed.

### Immunogenicity of the SF2a-TT15 Preclinical Batch in Mice

**Figure 4:**
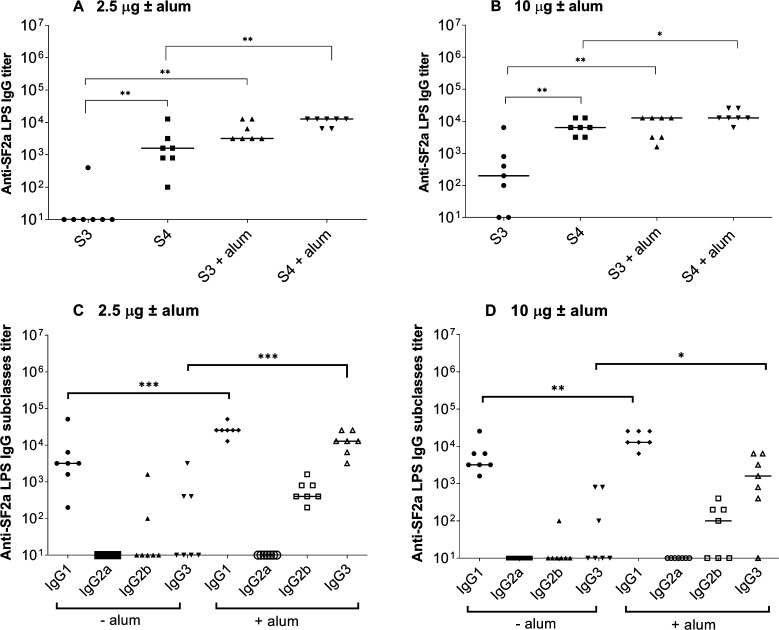
Immunogenicity of the SF2a-TT15 preclinical batch. Mice were immunized i.m. three times at a three-week interval, followed by a fourth injection one month later with an equivalent of 2.5 μg or 10 μg of carbohydrate per dose adjuvanted or not with alum. Anti-SF2a LPS IgG titers were measured by ELISA 7 days after the third (S3) and fourth (S4) injection (panels A and B). Anti-SF2a LPS IgG subclass titers (panels C and D) were determined on the day of the fourth injection. Mann–Whitney nonparametric *t* test: *p < 0.05; **p < 0.005; ***p < 0.0005. The Ab titer median value is indicated with a horizontal bar.

In the frame of process optimization to achieve SF2a-TT15, we showed that four injections in mice of a conjugate amount equivalent to 2.5 μg of oligosaccharide, as compared to three injections, slightly increased the anti-SF2a LPS IgG titer,^51^ thereby confirming original observations.^49^ Adjuvanting with aluminum hydroxide (alum, Al(OH)_3_) significantly increased the immunogenicity of SF2a-TT15, with a sustained anti-SF2a LPS IgG titer still observable at six months after the last injection.^51^ The immunogenicity of the preclinical batch was similarly assessed. The previously observed positive impact of a fourth injection and of alum, on the immunogenicity of SF2a-TT15 used at a dose of 2.5 μg of oligosaccharide was confirmed (Figure 4A). Similar results were obtained while using a higher dose, i.e., 10 μg of [AB(E)CD]_3_ (Figure 4B). Noticeably, increasing the dose had no significant impact on the anti-SF2a LPS IgG titer. For both doses, formulation with alum overcame the need for a fourth injection. The sustained response at six months post the last injection was confirmed for both doses (data not shown).

Regarding the IgG subclasses, SF2a-TT15 elicited predominantly an anti-SF2a LPS IgG1 response which significantly increased in the presence of alum for both doses. Of note, the specific IgG3 response that was very low upon immunization with the nonadjuvanted conjugate, was significantly increased with the adjuvanted one for both doses (Figure 4C,D). These findings show the immunogenicity of the SF2a-TT15 preclinical batch in mice and confirm the role of alum in potentiating the anti-SF2a LPS IgG response. In addition, data indicate that alum favors the induction of both SF2a-specific IgG1- and IgG3-mediated responses.

### Cross-Reactivity of the Antibodies Induced by the SF2a-TT15 Preclinical Batch toward Other *S. flexneriserotypes*

**Figure 5:**
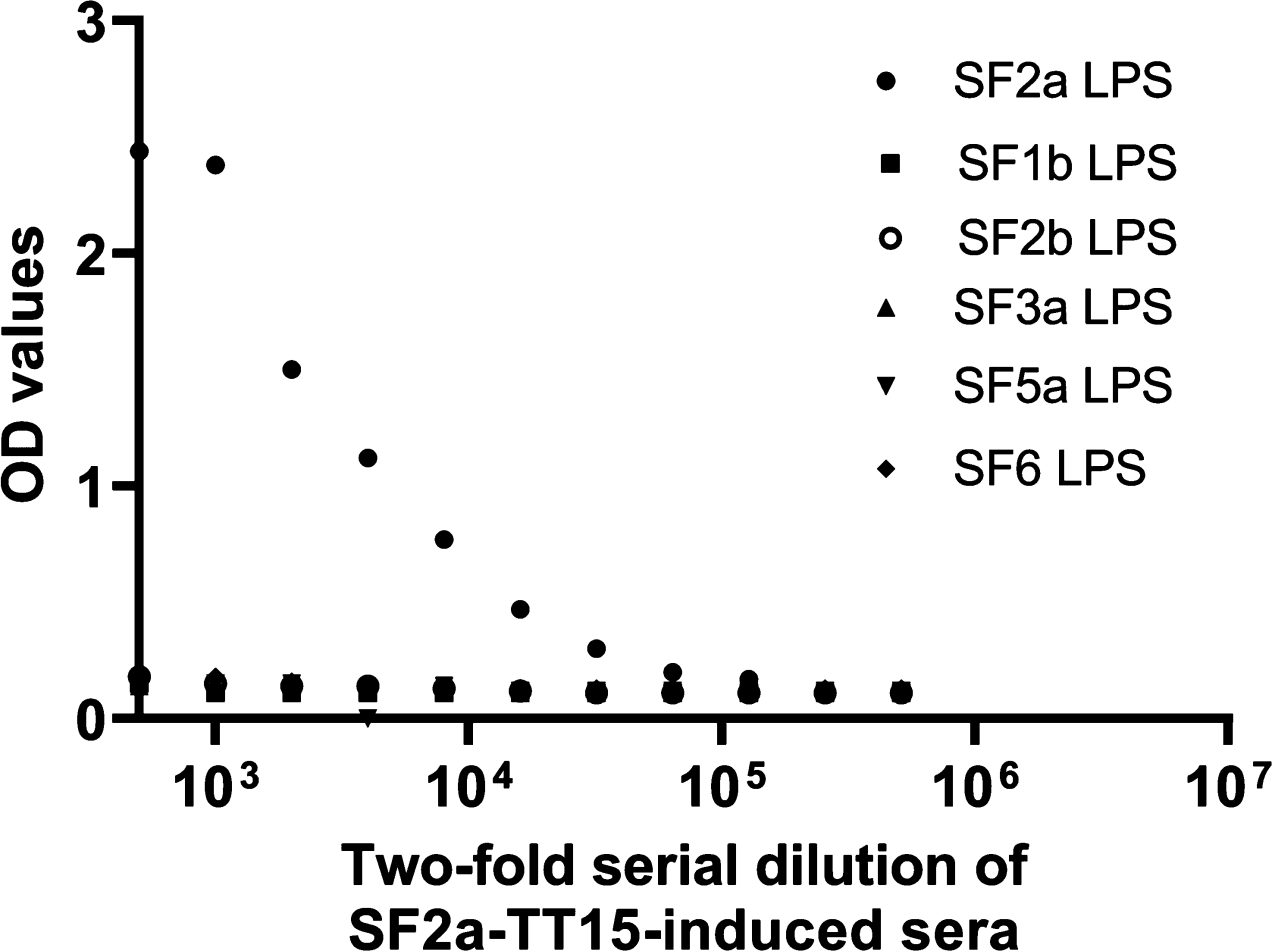
Cross-reactivity of the SF2a-TT15 preclinical batch-induced antibodies. Sera from seven mice immunized with the equivalent of 10 μg [AB(E)CD]_3_ per dose were pooled, diluted, and tested in ELISA toward a panel of LPSs purified from different *S. flexneri* serotypes strains. OD: optical density.

Sera from mice immunized twice with the adjuvanted equivalent of 10 μg [AB(E)CD]_3_ were tested in ELISA for their binding to LPS purified from SF1b, 2b, 3a, 5a, and 6 as compared to the SF2a LPS. Of note, for SF1b and SF6, two and three different strains, respectively, were used as a source of purified LPS. None of the tested LPSs were recognized (Figure 5), indicating that the murine antibodies induced by the SF2a-TT15 preclinical batch were specific for the homologous SF2a LPS among those tested.

### Bactericidal Activity of the anti-SF2a LPS IgG Abs Induced by the SF2a-TT15 Preclinical Batch

In previous reports, we used the mouse model of pulmonary infection to report the protective capacity of the anti-SF2a LPS IgG Abs induced by SF2a glycoconjugates.^49,51^ Considering that the serum bactericidal assay (SBA) was recently recognized as the “gold-standard” assay to assess the functionality of antibodies induced by *Shigella* vaccine candidates,^53^ the bactericidal antibody titer induced by the SF2a-TT15 preclinical batch was determined. For each tested condition, decomplemented sera pooled from seven immunized mice were used. For the 2.5 μg saccharide dose administered three times without and with alum, the SBA titer (mean value ± SD) was 4600 ± 280 and 16000 ± 500, respectively. The corresponding values for the 10 μg dose administered without and with alum were 10500 ± 500 and 27000 ± 3000, respectively. For both doses, the SBA titer increase observed in the presence of alum was in accordance with the increase of the anti-SF2a LPS IgG titer as compared to the nonadjuvanted conjugate (Figure 4). Similarly, the protective capacity of the SF2a-TT15-induced Abs was shown previously to be dependent on the Ab titer when measured in the murine model of pulmonary infection.^51^

### Recognition of a Panel of Clinical SF2a Isolates by the anti-SF2a LPS IgG Abs Induced by the SF2a-TT15 Preclinical Batch

The objective of a vaccine being able to protect against the largest diversity of circulating strains, we assessed the recognition of a panel of SF2a clinical isolates characterized at the French National Reference Center for *Enterobacteriaceae* (Institut Pasteur, Paris), upon recovery from stools of individuals with a diarrheal episode when back from traveling to different countries. The clinical isolates were compared to SF2a strain 454, the reference strain used for selecting the oligosaccharide hapten in the SF2a-TT15 conjugate.^40,49^

**Figure 6:**
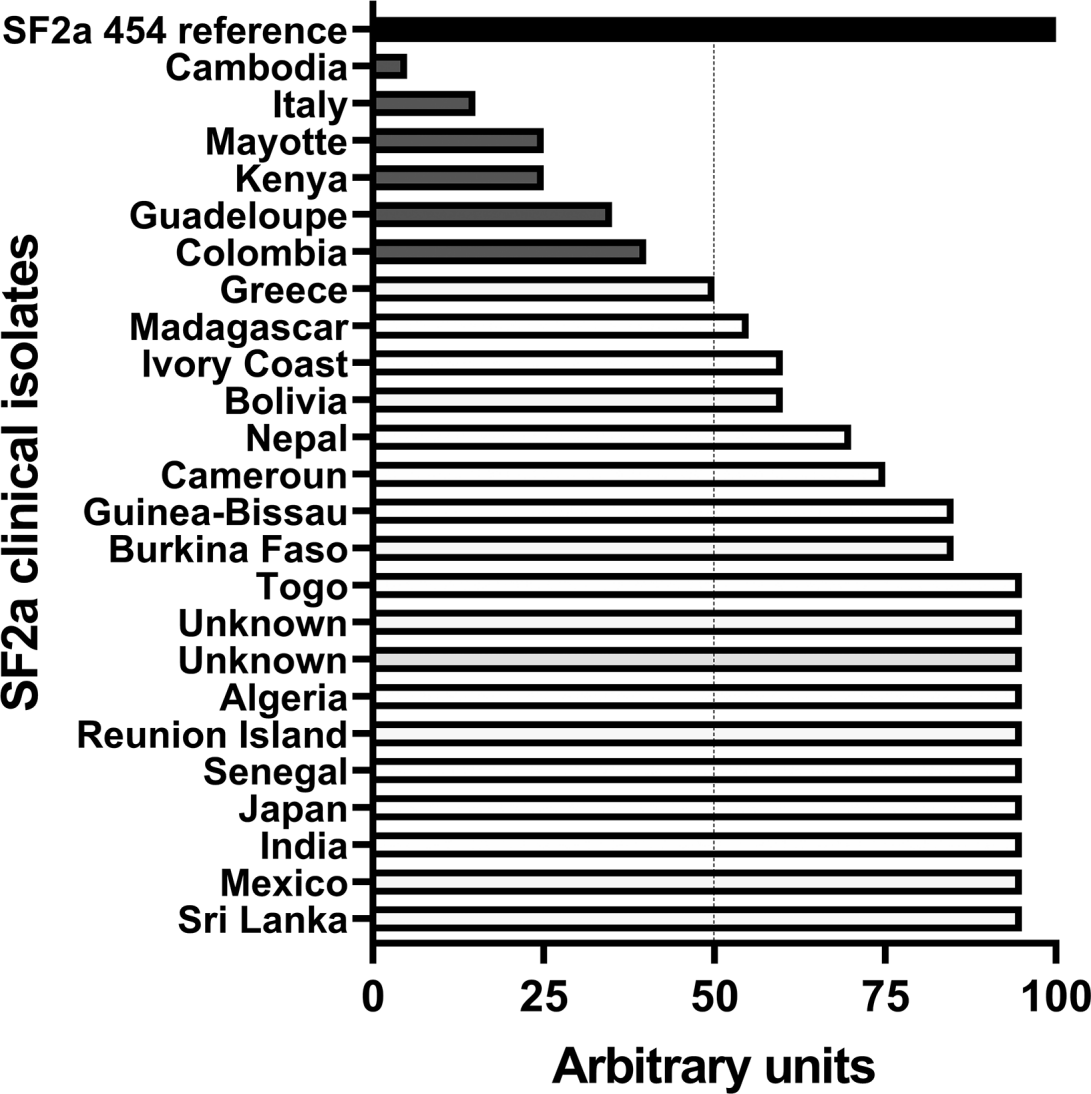
Recognition of a panel of 24 SF2a clinical isolates by SF2a-TT15-induced sera. Strain recognition was performed by FACS and arbitrary units defined considering a value of 100 for the reference strain. Data are representative of three independent experiments. FACS: fluorescence-activated cell sorting.

As shown in Figure 6, 18 out of the 24 tested strains were above 50% recognition as compared to SF2a 454, while six were below. The two lowest recognized clinical isolates from Italy and Cambodia were shown to express much less LPS as compared to the other strains (data not shown). These results show that the SF2a-TT15 preclinical batch gives rise to specific SF2a Abs recognizing a large diversity of SF2a circulating strains.

### SF2a-TT15 Toxicology Assessment

To provide data on the local and systemic toxicity and reactogenicity of the formulated preclinical SF2a-TT15 (10 μg carbohydrate per dose), either adjuvanted with Al(OH)_3_ or nonadjuvanted, male and female rabbits received four intramuscular (i.m.) injections at a 3 week interval. All rabbits were monitored for different parameters: body weight, food intake, body temperature (before, 4 and 24 h after each dose), hematology (red blood cell parameters, coagulation parameters, total or differential white blood cell counts), clinical chemistry, and macro- and microscopic examination of organs. The parameters were either not affected by the treatment or their analysis did not reveal any treatment related effects (not described). The Al(OH)_3_-adjuvanted SF2a-TT15 formulation showed mild to moderate widespread mixed inflammation at the injection site. The nonadjuvanted vaccine candidate showed localized mixed inflammation or mild widespread mixed inflammation at the injection site (Tables S2 and S3). For both groups, these effects subsided 17 days after the last injection.

It was concluded that the 10 μg carbohydrate-equivalent formulation of the synthetic glycan-based conjugate vaccine candidate, whether alum-adjuvanted or nonadjuvanted, was well tolerated and did not result in any signs of systemic toxicity in vaccine recipients. Additionally, SF2a-TT15 was immunogenic in rabbits (Figure S6) as it was shown to be in mice (Figure 4). Therefore, the evaluation of toxicity as described took into account the presence of the anti-SF2a LPS IgG response.

## Conclusions

The Gram-negative bacterium SF2a is the most prevalent *S. flexneri* serotype and the main cause of shigellosis. The need for a vaccine that would protect the youngest living in low-income settings against shigellosis was emphasized recently as *Shigella* was identified as a dominant cause of diarrheal disease in this population. Despite the attractiveness of synthetic glycans as vaccine components,^39,54,55^ limited access to chemically defined complex oligosaccharides has held up investigations on their potential use in the context of antibacterial vaccines. Herein, following up on the successful licensing of Quimi-Hib, we have described the GMP manufacturing of preclinical and clinical batches of SF2a-TT15, a synthetic carbohydrate conjugate vaccine candidate designed against endemic shigellosis. The scale-up of the original 40-step synthetic process^1,51^ was achieved successfully to reach a 100 mL production scale of the [AB(E)CD]_3_-TT conjugate complying with established critical standards, among which glycan loading and aggregation. This corresponded to a volumetric increase by a factor 625 of the microscale conjugation step, further demonstrating the established process robustness. As part of these developments, the use of TFF was proven feasible with conjugation kinetics and efficiency comparable to that seen at the microscale. The final high yielding production of both batches (76% and 80% for the SF2a-TT15 preclinical and clinical batches, respectively, with reference to the oligosaccharide precursor ^GMP^**2** and buffer exchanged ^GMP^TT (^GMP^**4**) provided several tens of thousands of doses of SF2a-TT15 (^GMP^**6**). The synthetic glycan-TT conjugate designed for vaccination against SF2a infection fulfilled all GMP criteria. The DS was formulated to achieve two vaccine doses (2 μg and 10 μg of glycan per injection, respectively). Whether alum-adjuvanted or nonadjuvanted, SF2a-TT15 passed all toxicology criteria and exhibited strong anti-SF2a immunogenicity in both mice and rabbits. The SF2a-TT15-induced antibodies are specific for the SF2a LPS and functional *in vitro*, exhibiting high anti-SF2a SBA titers. Moreover, they bound to a large diversity of SF2a circulating strains isolated from individuals diagnosed with shigellosis. It is noteworthy that many bacterial polysaccharides are diversely O-acetylated and that O-acetyl groups may compose a meaningful part of the immunodominant epitopes expressed at the surface of pathogenic bacteria.^56,57^ Yet, the extent to which O-acetylation contributes to the immunological properties of polysaccharide antigens is highly variable.^58,59^ Herein, the observed broad SF2a strain recognition substantiated our original observation,^60^ also underlined by others,^61,62^ that O-Ag O-acetylation does not play a major role in the antibody-mediated immunity to SF2a, despite the fact that SF2a strains are knowingly characterized by O-Ags featuring repeating units di-O-acetylated to various extents.^2−4^ These findings support the selection of the non-O-acetylated [AB(E)CD]_3_ glycan component in SF2a-TT15, thereby facilitating product manufacturing to a meaningful extent.^63,64^ Not the least, the implementation of a unique homogeneous chemically defined glycan hapten missing the naturally occurring labile substitutions also avoids challenging analytical issues and stability considerations otherwise of concern^65^ and therefore cost. The SF2a-TT15 formulations corresponding to both the 2 μg and 10 μg of glycan per vaccine dose were shown to be stable for at least 66 months. In particular, the free carbohydrate content fulfilled typical published specifications both at product release (<10%) and over vaccine shelf life (<25%).^64^ Despite the possible concern arising from reports on the sensitivity of antibody-drug conjugates featuring a thiol-maleimide linker to the chemical and structural dynamics at the conjugation site,^66^ our data suggest that spacer hydrolysis resulting from the retro-Michael addition of the formed thiosuccinimide^67^ remains in an acceptable range under formulation and storage conditions. Besides stability, concern stems from the possibility to generate meaningful levels of anti-spacer antibodies following immunization with glycan conjugates. In the worst case scenario, immunity diverges toward immunodominant epitopes present on the spacer resulting in poor antibody titers against the glycan component.^68,69^ We have previously stressed the absence of SF2a-TT15-induced detectable anti-linker antibodies in mice.^49^ Following up on the licensing of Quimi-Hib, issued from the conjugation of a maleimide-equipped polyribosylribitolphosphate hapten and thiolated TT,^70^ and adding to a remarkable hapten conjugation yield, far above the readily and consistently achievable 25–40% yield for glycoconjugate vaccine manufacture,^64^ the significant long-term stability of SF2a-TT15 promotes thiol-maleimide bioconjugation as one of the existing biorthogonal chemistries to explore further in the context of glycoconjugate vaccine development.^71,72^

This original glycoconjugate vaccine candidate was shown to be safe and well tolerated in healthy adults while inducing high titers of anti-SF2a LPS IgGs with bactericidal activity toward SF2a bacteria *in vitro*.^37^ The 10 μg saccharide dose, alum-adjuvanted or not, was demonstrated to be highly potent, inducing the highest IgG antibody titer after the first injection. No boosting effect followed the second and third injections. In contrast, for the adjuvanted 2 μg saccharide dose, a boosting effect of the second and third injections was observed in humans. Interestingly, mouse SBA titers might be considered as predictive of what will be induced in humans.^35^ In fact, high SBA titers were also measured in the human volunteers. Therefore, it is likely that the diversity of strain recognition shown here with the mouse sera might be extrapolated to human SF2a-TT15-induced sera. Indeed, both assays rely first on the capacity of the vaccine-induced antibodies to bind SF2a bacteria. The successful outcome of this first-in-human study complemented by robust preclinical data as disclosed herein, including long-term stability data demonstrating highly similar quality with the formulated conjugate administered in the phase 1, contributed to prompt further evaluation in humans of SF2a-TT15, a vaccine candidate elaborated from the understanding of the structural basis of the immune recognition of LPS-protective epitopes.^47,48,60^ Aiming at derisking product development,^73^ a Control Human Infection Model (CHIM) study^74^ will shed light on the protective capacity of SF2a-TT15 in naive adults (NCT0478022). Otherwise, an age-descending study in Kenya will assess the safety and immunogenicity of SF2a-TT15 in the target population, especially infants, in the field (NCT04602975).

This first detailed report on the GMP process of a synthetic carbohydrate–protein conjugate vaccine candidate targeting an infectious disease supports feasibility and strongly encourages further development in a domain, which is the subject of rapidly growing interest.

## Supporting Information Available

Additional information on the conjugation kinetics, impurity removal, and immunogenicity data, including toxicology study results. ^1^H NMR spectrum for oligosaccharide ^**GMP**^**2** (PDF)

## Dedication

^⊥^ This manuscript is dedicated in loving memory of Carolien Smitsman, for her insights and support on this project. We are forever grateful for her contributions.
