# Supplementary material for: The First-in-Human Synthetic Glycan-Based Conjugate Vaccine Candidate against Shigella
Source: ACS Cent Sci. 2022 Mar 17;8(4):449–60. doi: 10.1021/acscentsci.1c01479 (PMC9088300; doi:10.1021/acscentsci.1c01479)
Supplement: Supplementary file 1 — oc1c01479_si_001.pdf [file oc1c01479_si_001.pdf]

Supporting Information

The first-in-human synthetic glycan-based conjugate vaccine candidate against *Shigella*

Robert M. F. van der Put,*[a] Carolien Smitsman,[a†] Alex de Haan,[a] Martin Hamzink,[a] Hans Timmermans,[a] Joost Uittenbogaard,[a] Janny Westdijk,[a] Michiel Stork,[a] Olga Ophorst,[a] Françoise Thouron,[b] Catherine Guerreiro,[c] Philippe J. Sansonetti,[b,d#] Armelle Phalipon,[b§] and Laurence A. Mulard*[c]

[a] Intravacc, P.O. Box 450, 3720 AL Bilthoven, the Netherlands.

[b] Institut Pasteur, U1202 Inserm, Unité de Pathogénie Microbienne Moléculaire, 28 rue du Dr Roux, 75724 Paris Cedex 15, France.

[c] Institut Pasteur, Université de Paris, CNRS UMR3523, Unité de Chimie des Biomolécules, 28 rue du Dr Roux, 75724 Paris Cedex 15, France.

[d] Collège de France, Chaire de Microbiologie et Maladies Infectieuses, 11, place Marcelin Berthelot, 75005 Paris, France.

* *Corresponding authors: E-mail:* robert.van.der.put@intravacc.nl, laurence.mulard@pasteur.fr

I. Supplementary Tables S-2

II. Supplementary figures S-3

III. Methods S-6

REFERENCES S-9

I. Supplementary Tables

Table S1. Conjugation efficiency using precursors **2** and **5** of different qualities.

	Microscale				Intermediate scale	
Reagents	R&D**2** + R&D**5**		GMP**2** + R&D**5**		GMP**2** + GMP**5**	
**2:5** ratio (mol:mol)	**3:4** loading (mol:mol)	Conjugation efficiency (%) [a]	**3:4** loading (mol:mol)	Conjugation efficiency (%) [b]	**3:4** loading (mol:mol)	Conjugation efficiency (%) [c]
6.25	4.6	71	4.3	72	n.a.	n.a.
12.5	8.5	65	7.1	59	n.a.	n.a.
25	17	63	15	63	17 ± 1	62 ± 5
50	26	49	25	52	n.a.	n.a.

[a] Average results from two experiments. [b] Results from a single experiment. [c] Average results from three experiments.

Table S2. Toxicology results summary microscopic observations (subgroup 1, sacrificed 3 days after the last injection) using the pre-clinical batch of SF2a-TT15 (**3**:**4** loading (mol/mol): 19) versus placebo (20 mM TRIS, 150 mM NaCl).

Vaccine / Placebo		SF2a-TT15	Adjuvanted SF2a-TT15	Placebo	SF2a-TT15	Adjuvanted SF2a-TT15	SF2a-TT15
Animals	Gender	Male			Female		
	Number	5	5	5	5	5	5
Muscle, anterior thigh, right	Inflammation, mixed, localized (minimal/mild)	0	0/2	0	0	1/1	1
	Accumulation of swollen macro-phages (mild/mod-erate)	0	3/1	0	0	5/0	0
Muscle, anterior thigh, left	Necrosis moderate	0	3	0	0	3	0
	Inflammation, mixed, widespread (mild/moderate)	0	1/3	0	4/0	3/2	0

Table S3. Toxicology results summary microscopic observations (subgroup 2, sacrificed after 14 day recovery period) using the pre-clinical batch of SF2a-TT15 (3:4 loading (mol/mol): 19) versus placebo (20 mM TRIS, 150 mM NaCl).

Vaccine / Placebo		SF2a-TT15	Adjuvanted SF2a-TT15	Placebo	SF2a-TT15	Adjuvanted SF2a-TT15	SF2a-TT15
Animals	Gender	Male			Female		
	Number	3	3	3	3	3	3
Muscle, anterior thigh, right	Inflammation, mixed, localized (minimal/mild)	0	1/1	0	0	1/2	0
	Accumulation of swollen macro-phages (mild/mod-erate)	0	3/0	0	0	1/2	0
Muscle, anterior thigh, left	Necrosis moderate	0	1	0	0	1	0
	Inflammation, mixed, widespread (mild/moderate)	0	1	0	0	1	0

II. Supplementary figures

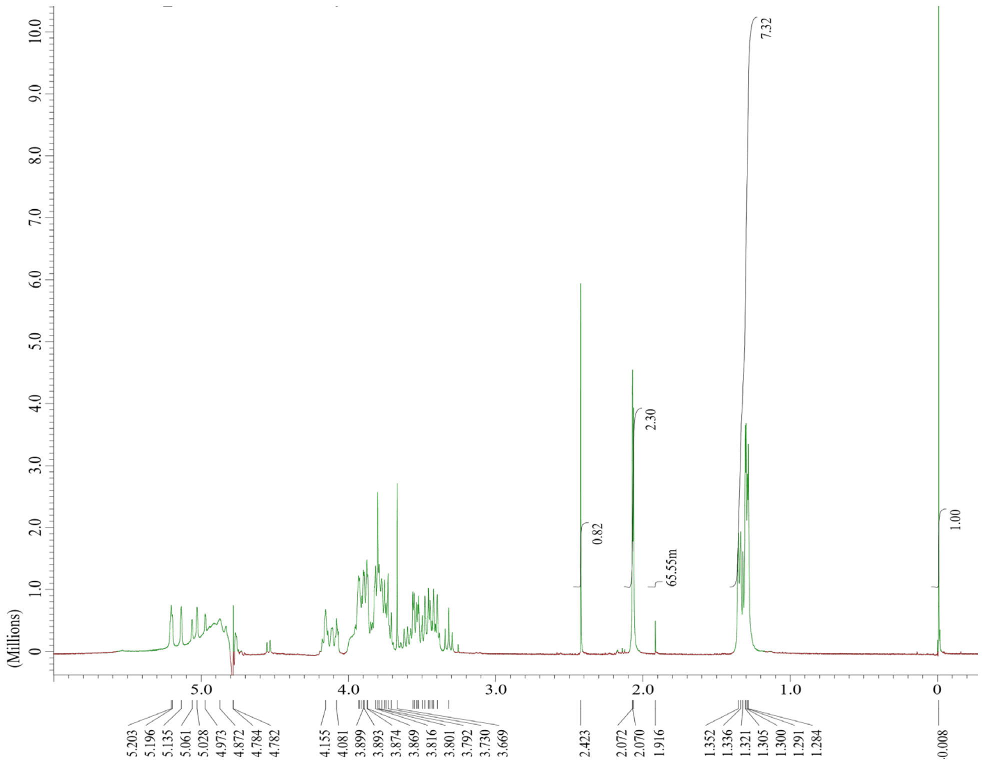

Figure S1. ^{1}H-NMR spectrum of conjugation-ready ^{GMP}**2** (D_2O, 400 MHz). x-axis: chemical shift (ppm), y-axis: signal intensity with reference to 3-(trimethylsilyl)propanoic acid (TMSP) as internal standard (*δ* -0.008 ppm). Extracted: *δ* 1.35-1.28 (m, 27H, H-6A, H-6B, H-6C), 2.07-1.91 (3s, 9H, H-NAc), 2.42 (s, 3H, H-SAc).

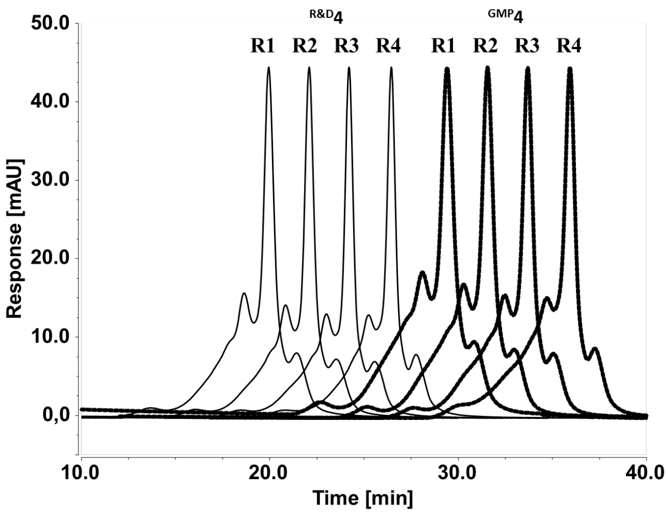

Figure S2. HPSEC analysis of conjugates (microscale experiments). x-axis: retention time (min), y-axis: response (mAU). UV signals were normalized for the monomer peak (highest peak in chromatogram) and an offset was applied to each individual trace to facilitate the overview. Black full trace: using $^{R\&D}$**2** and $^{R\&D}$**5**, black dashed trace: using ^{GMP}**2** and $^{R\&D}$**5**, R: **2**:**5** input ratio (R1: 6.25, R2: 12.5, R3: 25, R4: 50). **HPSEC method:** An UltiMate-3000 (Thermo Fisher Scientific) HPLC system was equipped with a SEC Guard column 100 (Wyatt Technology), SEC protein column 100 (Wyatt Technology), and SEC protein column 30 (Wyatt Technology) mounted in series (in this order). The eluent was 0.1 μm sterile filtered phosphate buffered saline (PBS) 10 mM, pH 7.2, and elution was performed at 1 mL/min (average backpressure 60 bar). All chromatographic parameters were calculated using the Chromeleon software (v. 7.1.2, SP 2, Thermo Fisher Scientific).

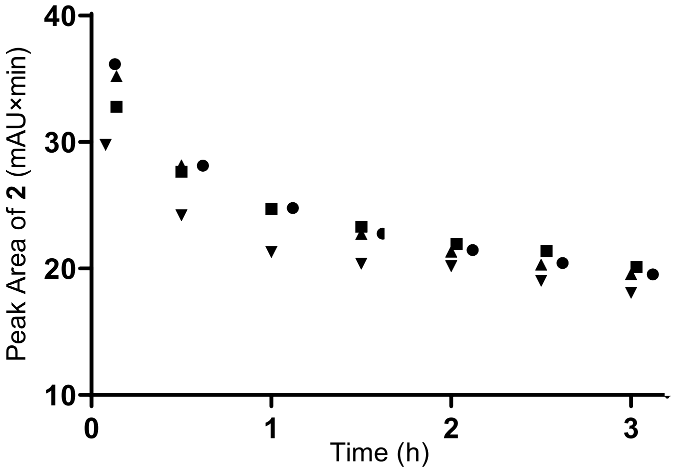

Figure S3. Conjugation kinetics for a **2:5** ratio (mol:mol) of 25 (HPSEC follow up), showing the decrease in peak area of **2** in time. x-axis: time (h), y-axis: Peak Area of $^{R\&D}$**2** entity (mAU×min) for microscale conjugation (●) and (■), and of ^{GMP}**2** at micro (▲) and intermediate (▼) scale. HPSEC: high performance size exclusion chromatography.

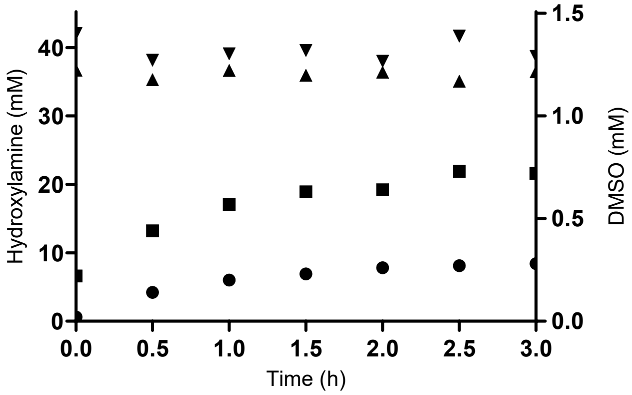

Figure S4. DMSO and hydroxylamine concentration during conjugation. X-axis: Time (h), y-axis: concentration (mM) quantified by NMR. (●) DMSO, LP screen filter; (■) open channel filter; (▲) hydroxylamine, LP screen filter; (▼) hydroxylamine, open channel filter.

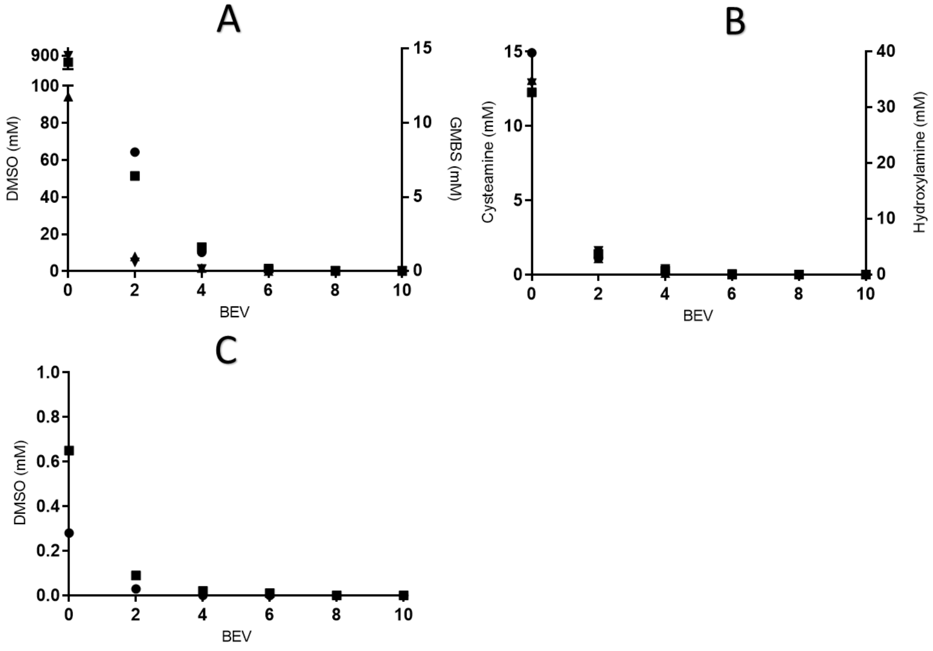

Figure S5. Follow up of impurity concentrations during processing and final buffer exchange at the intermediate scale. (**A**) DMSO and GMBS/GMBA concentrations during maleimide modification of **4** into **5,** ● DMSO LP screen ■ DMSO open channel ▲ GMBS LP screen ▼ GMBS open channel; (**B**) Cysteamine and hydroxylamine concentrations during SF2a-TT15 final buffer exchange, ● cysteamine LP screen ■ cysteamine open channel ▲ hydroxylamine LP screen ▼ hydroxylamine open channel; (**C**) DMSO concentration during SF2a-TT15 final buffer exchange● DMSO LP screen ■ DMSO open channel; (**A**, **B** & **C**) x-axis: buffer exchange volumes (BEV), y-axis: concentration (mM) quantified by NMR.

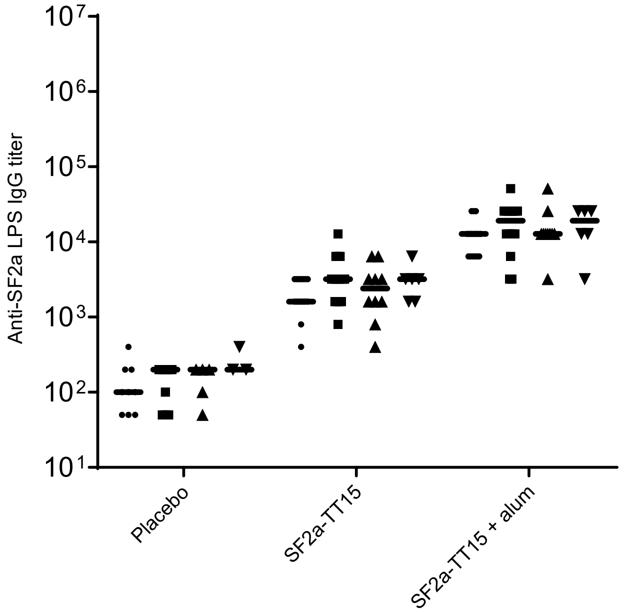

Figure S6. Immunogenicity of SF2a-TT15 preclinical GMP batch in rabbits. Groups of 8 rabbits were immunized i.m. with SF2a-TT15 non adjuvanted or alum-adjuvanted, or with placebo, four times at three weeks interval, *i.e.* Day 0, 21, 42, and 63. Blood samples were recovered at D42 (3 weeks after the 2nd injection, just before the 3rd injection), Day 63 (3 weeks after the 3rd injection, just before the 4th injection), and D66 (3 days after the 4th injection), and D77, 14 days after the 4th injection. The kinetics of anti-SF2a LPS IgG titers measured by ELISA is presented. Bars indicate the median of the antibody response. ● Day 42 ■ Day 63 ▲ Day 66 ▼ Day 77.

III. Methods

Safety statement: no unexpected or unusually high safety hazards were encountered during any of the methods, processes or assays.

Chemicals: all chemicals used and presented in this manuscript were of the highest possible available purity and complying with GMP regulations where needed.

General methods BCA assay, TNBS assay, Anthrone assay, High Performance Size Exclusion Chromatography (HPSEC), buffer exchange, concentration and conjugation kinetics determination were performed as described,[1] however with the addition that the methods were validated for GMP application. NMR analysis was run on a FT-NMR 400 (JEOL) in pre-saturation mode and signal intensity with reference to 3-(trimethylsilyl)propanoic acid (TMSP, Aldrich). Osmolality was measured using a calibrated Osmomat 3000 (Gonotec). When applicable, samples were prediluted using Milli-Q water (Millipore). The pH was measured using a calibrated FE20 Basic FiveEasyTM pH-meter (Mettler Toledo) and an Inlab Nano pH electrode (Mettler Toledo). ^{GMP}TT was acquired through the open market, from Bilthoven Biologicals (Bilthoven, The Netherlands). It was released for production based on review of the certificate of analysis and compliance to internal specifications in terms of concentration (> 1500 LF). The ready-for conjugation ^{Tox}**2** and ^{GMP}**2** were acquired through the open market, from Sanofi CEPiA (Antony, France). They were released for production based on review of the certificate of analysis, purity assessment by ^{1}H-NMR and HPAEC-PAD, and compliance to pre-defined specifications relevant for drug substance (DS) intermediates. Reagents and solvents were from the highest quality compliant with GMP regulations.

Microscale bioconjugation The microscale bioconjugation assays (0.16 mL each), applying ^{GMP}**2**:$^{R\&D}$**5** ratios of 6.25, 12.5, 25 and 50 were performed as described earlier.[1]

Intermediate-scale bioconjugation The [^{GMP}**2** + ^{GMP}**5**] bioconjugation at intermediate-scale (1.0 mL) was performed essentially according to the protocol used for the microscale conjugation, respecting the reaction conditions.[1] However, in this case ^{GMP}TT was used and only the ^{GMP}**2**:^{GMP}**5** ratio (mol:mol) of 25 was investigated in triplicate. In short, $^{R\&D}$TT modified with GMBS (Thermo Fisher Scientific, 18.8 mg, 125 nmol) in 0.1 M Phosphate buffer, 5 mM EDTA (in-house), pH 6.0 (18.8 mg/mL, 1.0 mL each) was reacted with ^{GMP}**2** (8.1 mg, 3.1 µmol, 25 equiv.) in 0.1 M Phosphate buffer, 5 mM EDTA, pH 6.0 (64.7 mg/mL, 125 µL). Hydroxylamine (Thermo Fisher, 3.3 mg, 46.9 µmol) in 0.1 M Phosphate buffer, 5 mM EDTA, pH 6.0 (125 µL, 26.1 mg/mL) was added in a 15:1 ^{GMP}**2** molar ratio. Conjugation was performed at ambient temperature for 180 min, with final protein concentration at 15 mg/mL. Conjugation progress was monitored by HPSEC (injections at 30 min intervals). After 3 h, Cysteamine·HCl (Sigma Aldrich, 2.3 mg, 20.0 µmol, 160 equiv.) in water for injection (WFI, 125 µL, 18.2 mg/mL) was added to all reaction mixtures. Final products were all purified (30 kD Amicon Ultra 15, Merck Millipore, UFC9030) using 20 mM TRIS buffer pH 7 (in-house).

Scale-up study on impurity removal Both scale-up and impurity removal studies were essentially performed similar to the GMP production of the preclinical batch (see below). However, during these studies only buffers and excipients were used. Additionally, changes relevant to the quantities of BEV occurred during purification (see main text).

Evaluation of process performance for two different filters TT maleimide activation was mimicked by 30 minutes of recirculation through the Cogent micro (Merck Millipore), the process time for reaction completion. The filters did not show any apparent interaction with DMSO, GMBS or GMBA. Both DMSO and GMBS/GMBA were removed efficiently (≥ 99.9%) after 8 and 6 buffer exchange volumes (BEV), for the LP screen and open channel filters, respectively (Figure S2A). Filtrate flow, inlet pressure, outlet pressure and trans membrane pressure (TMP) remained constant for both filters during the entire process (data not shown). Similarly, no interaction was observed between hydroxylamine and the filters during the 3 h recirculation simulating the conjugation reaction (Figure 4). In contrast, DMSO concentrations increased in time, where the open channel showed twice the amount of DMSO released from the filter compared to the LP screen filter (Figure 4). According to the manufacturer, this phenomenon was not uncommon, since DMSO was held up at the membrane surface during prior process steps in the presence of high concentrations of DMSO (6.3% v/v). This effect could be minimized decreasing the feed volume to membrane area ratio. However, it was decided to evaluate further removal of DMSO during following process steps. During simulation of the capping reaction, cysteamine concentrations remained constant during the 30 min recirculation. At the start of the final buffer exchange, cysteamine was already below the maximum tolerated concentration (518 mM, Table 2), however, more than 99.9 % was removed after 6 BEV (Figure S2B). Hydroxylamine was removed to below the maximum allowed concentration of 12 mM after 2 BEV, and more than 99.9 % after 8 BEV (Figure S2B). As suggested, DMSO extracted from the filter during conjugation, was removed to below detection limit at 4 BEV for the LP screen and 8 BEV for the open channel during final purification (Figure S2C). Final concentration of the TRIS formulation buffer (20 mM) was already acceptable, 18 mM, after 4 BEV for both filters (data not shown).

GMP production of a preclinical batch The entire production of the preclinical batch was performed in the reaction vessel of the Cogent µ (Millipore) equipped with a Sius-LSn TFF cassette (Repligen, TangenX, LP Screen channel, low binding mPES, 30 kD, 0.01 m^2). ^{GMP}TT (^{GMP}**4**, 1925 mg, 12.8 µmol) of the commercial solution (7.7 mg/mL, 250 mL) was concentrated to 100 mL and purified using five BEV (0.1 M HEPES buffer, pH 7.8, in-house) to give ^{GMP}TT (16.0 mg/mL, 1600 mg, 10.7 µmol). A solution of GMBS (478.3 mg, 1.7 mmol, 160 equiv., Thermo Fisher Scientific) in DMSO (6.7 mL, 71.7 mg/mL, Sigma Aldrich) was added and the reaction mixture was stirred for 30 min at ambient temperature. The modified ^{GMP}TT was purified using five BEV (0.1 M Phosphate buffer, 5 mM EDTA, pH 6.0, in-house) and concentrated to a final volume of 80 mL to reach $^{GMP}TT_{Mal}$ (^{GMP}**5**, 18.8 mg/mL, 1,500 mg, 10.0 µmol). $^{GMP}[AB(E)CD]_3$-SAc (^{GMP}**2**, 647 mg, 250.0 µmol, 25 equiv.) in 0.1 M Phosphate buffer, 5 mM EDTA, pH 6.0 (64.7 mg/mL, 10 mL) and Hydroxylamine·HCl ($^{GMP}[AB(E)CD]_3$-SAc x 15 equiv., 260.6 mg, 3750 µmol, Thermo Fisher) in 0.1 M Phosphate buffer, 5 mM EDTA, pH 6.0 (26.0 mg/mL, 10 mL) were added. After stirring for 180 min at ambient temperature, Cysteamine·HCl ($^{GMP}TT_{Mal}$ x 160 equiv., 182 mg, 1600 µmol, Sigma Aldrich) in 0.1 M Phosphate buffer 5 mM EDTA pH 6.0 (18.2 mg/mL, 10 mL) was added and the reaction mixture was stirred for 30 min at ambient temperature. Final purification was performed by 10 BEV (20 mM TRIS·HCl buffer, pH 7.0, in-house), after which the product was 0.22 µm filtered (Pall), analysed, and stored at 2 - 8 °C. Based on the amount of the starting ^{GMP}**2** (95.5% pure as measured by quantitative ^{1}H-NMR), the SF2a-TT15 conjugate was obtained in 76% corrected yield. Formulation of the 2 and 10 µg doses was achieved by dilution of the DS using 20 mM TRIS·HCl 150 mM NaCl pH 7.0 (in-house) to their respective carbohydrate concentrations – 8 µg/mL and 40 µg/mL, respectively – before final fill and finish.

GMP production of a clinical batch of SF2a-TT15 The protocol was identical to that described for the preclinical batch, except that the bioconjugation step involved the GMP compliant glycan precursor. Based on the amount of the starting ^{GMP}**2** (647 mg, 250.0 µmol, 25 equiv., 94.7% pure as measured by quantitative ^{1}H-NMR), the SF2a-TT15 conjugate was obtained in 80% corrected yield, which was very similar compared to the preclinical batch. Formulation of the 2 and 10 µg carbohydrate equivalent doses was as described above.

Identity ELISA Identification of SF2a specificity in SF2a-TT15 vaccine was performed as follows. A high binding microtiter plate (Greiner) was coated overnight at room temperature with horse anti-TT antibodies (Bbio, the Netherlands) in a concentration of 0.6 IU/mL in carbonate buffer pH 9.6. The plate was washed with tap-water containing 0.05% Tween 80 (Merck). Series of negative control (TT; Bbio) and low and high dose vaccine (200 ng/mL carbohydrate each) twofold diluted in assay buffer (0.01 M PBS containing 0.05% Tween 80 and 0.5 % protifar (Nutricia, the Netherlands)) were added to the plate and incubated at 37 °C for 2 h. After washing with tap-water/Tween (0.05%) the plate was incubated at 37 °C for 2 h with an anti-SF2a specific IgG mAb (Institut Pasteur) in a 1/2000 dilution in assay buffer. The plate was washed as above and 5000 x diluted Horseradish peroxidase (HRPO)-conjugated goat-anti mouse IgG (Southern Biotech) in assay buffer was added to the plate, followed by incubation at 37 °C for 1.5 h. The plate was washed and 3,3′,5,5′-tetramethylbenzidine (TMB) microwell peroxidase substrate (KPL) was added. After 10 min the reaction was stopped by addition of 0.2 M aq. H_2SO_4 (Sigma Aldrich) and absorbance at 450 nm was measured. Samples were positively identified if the average extinction of the first three dilutions of eleven dilutions of a twofold dilution series of the sample is equal to or higher than three times the average extinction of the first three matching dilutions of the negative control.

HPAEC-PAD-measured carbohydrate total content and free carbohydrate in the SF2a-TT15 conjugate bulks and formulations All analyses were performed on an ICS-3000 (Thermo Fisher Scientific) HPLC system equipped with an AminoTrap guard (4x50 mm, Thermo Fisher Scientific) and a CarboPac PA10 column (4x250 mm, Thermo Fisher Scientific). A standard curve was constructed (0 – 100 nmol repeating unit) consisting of Rha*p*, Glc*p*NAc and Glc*p* (3:1:1) and fucose (Fuc*p*, 25 nmol) (All, Sigma Aldrich), which was used as an internal standard. The method was validated for GMP applications. The total carbohydrate content was measured as follows. SF2a-TT15 conjugate formulations (0.5 mL) containing carbohydrates (8 and 40 µg/mL corresponding to the 2 and 10 µg carbohydrate content per vaccine dose, respectively), whether in their free form or conjugated, were hydrolyzed to their respective monosaccharides using 0.5 mL 4 M aq. TFA (121 °C, 60 min, Biosolve). Free carbohydrate content was measured as follows. SF2a-TT15 conjugate formulations containing carbohydrates (8 and 40 µg/mL corresponding to the 2 and 10 µg carbohydrate content per vaccine dose, respectively) were passed through 30 kD-filters. Sample filtrates were hydrolyzed as above. The hydrolyzed samples and standards were pre-diluted using 20 mM TRIS·HCl pH 7.0 (in-house) and eluted using 18 mM aq. NaOH (in-house) isocratic conditions. All chromatographic parameters were calculated using the Chromeleon software (v. 7.1.2, SP 2, Thermo Fisher Scientific).

Toxicology study in rabbit The study design comprised six groups of 8 animals (SPF quality New Zealand White albino rabbits, app. 13 weeks old, equal number of male to female). Group 1 received the conjugate vaccine (10 µg dose, 0.5 mL), group 3 received the vehicle control treatment (20 mM TRIS·HCl, 150 mM NaCl buffer, pH 7.0, 0.5 mL) and group 2 received the adjuvanted conjugate vaccine (10 µg dose, 0.5 mL containing $Al(OH)_3$ (alum, 1.36 mg/mL, Brenntag Biosector, Alhydrogel 2% Ph.Eur.)). The placebo or vaccine formulations were administered within 1 hour after preparation. Each animal received four i.m. injections each at a different site at three week intervals (on day 0, 21, 42 and 63). Blood samples were recovered at day 42, 63, 66 and 77 and kept at -20 °C. Each group was subsequently divided into two subgroups. Rabbits in the first groups (5 males and 5 females) were sacrificed three days after the last immunization (day 66). Members of the second groups (3 males and 3 females) were sacrificed after a 14 day recovery period post the last injection (day 80). The welfare of the animals was maintained in accordance with the general

principles governing the use of animals in experiments of the European Communities (Directive 2010/63/EU) and Dutch legislation (The revised Experiments on Animals Act, 2014).

Immunogenicity study in rabbits The glycoconjugate-induced anti-LPS IgG response specific for SF2a LPS was measured by ELISA using LPS purified from the SF2a 454 strain as previously described.[2] Briefly, purified SF2a LPS in 0.04 M carbonate buffer pH 9.6 (2.5 µg/mL, 100 µL) was added per ELISA plate well and the plates were incubated overnight at 2-8 °C. After washing the wells with Tween 80 0.05%, blocking was performed by incubating the plates for 30 min at 37 °C with PBS-BSA 1% (100 µL) followed by washing with Tween 80 0.05%. Then, rabbit sera (originating from the toxicology study, see above) in PBS-BSA 1% was added in a twofold dilution series and the plates were incubated for 1 h at 37 °C. After washing with Tween 80 0.05%, anti-rabbit IgG alkaline phosphatase-labeled conjugate (Sigma-Aldrich) was added to each well at a dilution of 1/20000 in PBS-BSA 1% (100 µL). The plate was covered with aluminum foil and incubated at rt for 1 h. The reaction was stopped by adding 1.5 M aq. NaOH (50 µL) to each well. The IgG Ab titer was defined as the last dilution of serum giving an OD value (λ = 405 nm) twice that of the OD value obtained with the pre-immune serum.

Immunogenicity study in mice SF2a-TT15, adjuvanted or not with alum, was administered to seven week-old Balb/c mice (Janvier Labs, France) i.m. at multiple sites with 2.5 or 10 µg equivalent of $[AB(E)CD]_3$ per dose in 0.5 mL of TRIS·HCl 20 mM pH 7.2. Alum-adjuvanted SF2a-TT15 was obtained by mixing v/v the conjugate with aluminum hydroxide (Alhydrogel, Brenntag, Denmark) at a concentration of aluminum of 1.4 mg/mL (corresponding to a dose of 0.35 mg of aluminum (Al^{3+}) per mouse/per injection) in TRIS·HCl 20 mM pH 7.2 for 30 min at ambient temperature with rotor shaking. Three injections were performed at three week-interval, and a fourth one, one month later. Blood samples were recovered one week after each injection and at six months after the 4th injection. Seven mice were used per group. All of the mice experiments were approved by the Institut Pasteur Animal Use Committee.

Measurement of the anti-SF2a IgG antibody response The glycoconjugate-induced anti-LPS IgG response specific for SF2a LPS was measured by ELISA using LPS purified from the SF2a 454 strain as previously described.[2] Briefly, 2.5 µg of purified SF2a LPS was coated per ELISA plate well in PBS and incubated at 4 °C overnight. After washing the wells with PBS-Tween 20 0.01%, saturation was performed by incubating the plate for 30 min at 37 °C with PBS-BSA 1%. Then, serial dilutions of mouse sera in PBS-BSA 1% were incubated for 1 h at 37 °C. After washing with PBS-Tween 20 0.01%, anti-mouse IgG alkaline phosphatase-labeled conjugate (Sigma-Aldrich) was used as secondary antibody at a dilution of 1/5000. The IgG titer was defined as the last dilution of serum giving rise to twice the OD value (λ = 405 nm) obtained with pre-immune serum.

SBA assay Sera from mice collected after the 3rd or the 4th immunization with either 2.5 or 10 µg $[AB(E)CD]_3$ equivalent, adjuvanted or not with alum, were pooled per group and heat-inactivated. The SF2a 454 strain, used as reference strain, was grown in Trypto-Casein-Soy (TCS) medium to log-phase (OD_{600nm}: 0.2), diluted 1/30000 in SBA buffer (50 mM phosphate buffer, 0.5% BSA) to $3x10^3$ colony forming units (CFU)/mL and distributed into sterile polystyrene U bottom 96-well microliter plates (20 µL/well). Heat-inactivated sera were serially diluted 2-fold, and added to each well (75 µL/well). Baby Rabbit Complement (BRC, Cederlane) diluted twice in SBA buffer was then added to each plate (50 µL/well), completed to a final volume of 150 µL per well with SBA buffer. Incubation of bacteria with complement (in the absence of serum sample) was used as control. After 2 h at 37 °C, for each well, 100 µL out of the 150 µL were plated on TCS-agar plates. CFU counting was performed after 24 h of incubation at 37 °C. SBA titer was determined as the serum dilution required to obtain 50% CFU reduction as compared to control. Both samples and control were tested in duplicates and two independent experiments were performed.

Recognition of a set of SF2a clinical isolates by SF2a-TT15-induced murine sera The SF2a clinical isolates were obtained from the French National Reference Center for *Escherichia coli, Shigella* and *Salmonella* (Institut Pasteur, Paris, France). They were isolated and characterized from stools of individuals developing diarrhea when back from travelling to the indicated country. After isolation on Congo red plates, one single colony for each clinical isolate and the SF2a 454 strain, used as the reference strain to identify $[AB(E)CD]_3$ as the SF2a-TT15 hapten were grown overnight in TCS (Trypto-Casein-Soy) medium at 37 °C with shaking. Then, 5 mL of each bacterial culture was centrifuged at 5000 rpm for 5 min and the pellet was suspended in PBS/BSA 0.5% to a concentration of 10^7 CFU/mL. After a washing step using PBS/BSA 0.5%, bacteria resuspended in 1 mL of PBS/BSA 0.5% were incubated with 200 µL of mouse serum samples diluted 1:20. After 30 min incubation at 37 °C, two PBS/BSA 0.5%-washings were performed, and the bacterial pellet resuspended in 1 mL of the same buffer was incubated with anti-mouse IgG Alexa Fluor 488-labeled secondary antibody (Thermo Fisher) at a dilution of 1:2,000. After 30 min incubation at 37 °C in the dark, and two PBS-washings, bacteria resuspended in PBS (1.0 mL) were analyzed by flow cytometry (Attune, Thermo Fisher). Controls using AF 488-only labelled bacteria, and bacteria incubated with pre-immune sera, were used to account for background fluorescence and non-specific binding of mouse IgG, respectively. Results were analyzed using FlowJo 10.3.

Statistical analysis For statistical significance while comparing groups of mice, an unpaired *t* test was performed. *, **, and *** indicate a p value < 0.05, < 0.005, and < 0.0005, respectively.

REFERENCES

1. van der Put, R. M.; Kim, T. H.; Guerreiro, C.; Thouron, F.; Hoogerhout, P.; Sansonetti, P. J.; Westdijk, J.; Stork, M.; Phalipon, A.; Mulard, L. A., A synthetic carbohydrate conjugate vaccine candidate against shigellosis: Improved bioconjugation and impact of alum on immunogenicity. *Bioconjugate Chem.* **2016,** *27*, 883-892.
2. Phalipon, A.; Costachel, C.; Grandjean, C.; Thuizat, A.; Guerreiro, C.; Tanguy, M.; Nato, F.; Vulliez-Le Normand, B.; Belot, F.; Wright, K. et al, Characterization of functional oligosaccharide mimics of the *Shigella flexneri* serotype 2a O-antigen: implications for the development of a chemically defined glycoconjugate vaccine. *J. Immunol.* **2006,** *176*, 1686-1694.
